# Impact of COVID-19 on antimicrobial stewardship activities in Italy: a region-wide assessment

**DOI:** 10.1186/s13756-024-01407-3

**Published:** 2024-05-09

**Authors:** Costanza Vicentini, Silvia Corcione, Giuseppina Lo Moro, Alessandro Mara, Francesco Giuseppe De Rosa, Carla Maria Zotti, on behalf of the collaborating group “Unità Prevenzione Rischio Infettivo (UPRI), Regione Piemonte”, Fabrizio Bert, Cesare Bolla, Valentina Blengini, Roberta Broda, Francesco D’ Aloia, Francesco Di Nardo, Gerolamo Farrauto, Mauro Franco, Scipione Gatti, Franca Gremo, Agostino Maiello, Barbara Mitola, Domenica Morabito, Aida Muca, Orietta Ossola, Alessandro Paudice, Paolo Pellegrino, Claudio Plazzotta, Maurizio Salvatico, Paola Silvaplana, Carlo Silvestre, Pasquale Toscano, Valentina Venturino

**Affiliations:** 1 Department of Public Health and Pediatrics, University of Turin, Via Santena 5 bis, Turin, 10126 Italy; 2 Department of Medical Sciences, Infectious Diseases, University of Turin, Turin, Italy

**Keywords:** Antimicrobial stewardship, Quality indicators, Antimicrobial usage, Antimicrobial resistance, Quality improvement, Italy, COVID-19, Pandemic

## Abstract

**Background:**

In the region of Piedmont, in Northern Italy, formal monitoring of antimicrobial stewardship (AMS) programs has been in place since 2012. The objective of our study was to provide an updated assessment of AMS programs operating in our region, and to assess the impact of the COVID-19 pandemic on stewardship activities.

**Methods:**

A retrospective observational study was conducted to investigate AMS programs implemented in acute-care trusts participating in a broader healthcare-associated infections and antimicrobial resistance (AMR) prevention and control program, promoted by the regional health department. Within this program, structure, process, and outcome indicators of AMS programs were investigated, using a previously developed scoring system. Differences between scores prior to (2019) and during the pandemic (2021) were assessed. Linear regression was used to assess whether the 5-year trends (2017–2021) in outcome measures in relation to structure and process scores were statistically significant. Compound annual growth rates (CAGR) for each outcome were calculated to illustrate changes in outcome rates over time.

**Results:**

All public trusts in the Region (20) and a small number of private institutions (3) provided data for this study. A modest, non-significant improvement was found for 2021 structure, process, and total scores compared to respective 2019 scores. A significant improvement was found concerning the definition of a formal mission statement, whereas significantly less trusts included monitoring adherence to antimicrobial policy or treatment guidelines in their programs. Overall consumption of antibiotics for systemic use saw an increase in 2021, with 2021 recording the highest median overall consumption compared to all previous years considered in this study. Methicillin-resistant *Staphylococcus aureus* (MRSA) and carbapenem-resistant enterobacteria (CRE) rates decreased over the 5-year period. Significant downwards trends in MRSA rates were identified for high-outlier structure and process groups.

**Conclusions:**

Results of this study suggest AMS programs in Piedmont were not set back following the pandemic. This outcome was possible thanks to well-established programs, coordinated within a regional framework. Continued efforts should be dedicated to supporting AMS programs and contrasting AMR, even when the focus is shifted towards other public health emergencies.

## Background

Antimicrobial resistance (AMR) is a serious and urgent public health issue with wide-ranging effects on human health, animal welfare, and the condition of our ecosystem as a whole [1, 2]. Recent estimates suggest that the disease burden caused by antimicrobial resistance (AMR) is comparable to, or potentially surpasses, the combined impact of HIV and malaria. The 4.95 million fatalities caused by AMR in 2019 alone highlight the urgent need to address this growing danger to global health [3]. Overuse and misuse of antibiotics have been identified as major drivers of AMR. Hence, by implementing antimicrobial stewardship (AMS) programs and effectively reducing antimicrobial consumption, the risk of AMR can be significantly mitigated [4]. AMS programs have been established to enhance patient outcomes, ensure safety, and mitigate AMR while also curbing healthcare costs through the promotion of prudent and responsible use of antibiotics [5].

The situation in Italy in terms of AMR rates and antibiotic consumption is particularly critical. Italian AMR rates are among the highest in Europe, and even though decreasing trends in antibiotic consumption have been registered, both human and animal consumption remain higher than the European average [6, 7]. Consumption patterns also exhibit noteworthy regional variations, emphasizing the importance of developing strategies tailored to the local context [7]. 

To address these issues, a National action plan to contrast AMR (PNCAR) was published in 2017 and updated in 2022 [8, 9]. The PNCAR defines a roadmap for national, regional and local institutions to follow in order to contain AMR, and outlines strategies, indicators and targets in line with European and international plans [10]. The challenges posed by AMR in Italy underscore the critical need for concerted efforts to address this issue and implement effective strategies to combat the rise of AMR infections, highlighting the critical importance of effective AMS programs in our country [11].

The diversion of economic resources and healthcare personnel to address the Coronavirus disease 2019 (COVID-19) pandemic has resulted in the disruption or interruption of AMS programs and other non-primary health objectives. In Lombardy, a northern region of Italy neighboring Piedmont, factors such as hospital overcrowding and low healthcare worker-to-patient ratios were identified as potential reasons for the disruption of AMS activities, which were associated with increases in the use of antimicrobials and outbreaks of multidrug-resistant organisms [12].

In the region of Piedmont, formal monitoring of AMS programs has been in place since 2012, and surrogate outcome measures pertinent to AMS programs have been systematically recorded since 2017 [13]. The objective of our study was to provide an updated assessment of AMS programs operating in our region, and to assess the impact of the COVID-19 pandemic on stewardship activities.

## Methods

### Study design and data collection

A retrospective observational study was conducted to investigate AMS programs implemented in acute-care trusts in Piedmont, northern Italy. All public trusts participate in a broader healthcare-associated infections (HAI) and AMR prevention and control program, promoted by the regional health department and coordinated by the University of Turin. Through this regional program, data on indicators of HAI and AMR prevention and control activities are collected annually. Regional indicators reflect national prevention and action plans [8, 14]. Participation is mandatory for public trusts (18 in the region, totaling 49 hospitals), however private and not-for-profit hospitals can also participate, on a voluntary basis.

Within this program, structure, process, and outcome indicators of AMS programs were investigated. The development of the indicator system was previously described in detail; [13] for this study, data referring to the year 2021 were compared to 2019 data (pre-pandemic). Briefly, characteristics of AMS programs are assessed through 5 structure and 6 process indicators based on international guidelines and reviewed by a multidisciplinary expert panel [15, 16], with 0 points representing the lowest score and 10 points the highest score for each category of indicators. Table 1 summarizes structure and process indicators and respective scores. One response per trust was assessed, as AMS programs are often coordinated centrally within a trust.

**Table 1:** Quality indicators for the assessment of antimicrobial stewardship (AMS) programs operating in acute-care trust of the region of Piedmont, Northern Italy, and median scores for the years 2019 vs. 2021 (*N* = 23)

	2019 score	2021 score	*P* value^a^
**Structure indicators, median (range)**			
AMS team	2 (0–2)	2 (0–2)	0.783
Accountability	1 (0–2)	1 (1-2)	0.617
Mission statement	2 (0–2)	2 (1-2)	**0.035**
AMS policies	1 (1-2)	1 (0–2)	0.739
Microbiological laboratory quality management	0 (0–1)	0 (0–2)	0.414
*Overall structure indicator score*	*6* (3-9)	*7* (2-9)	*0.396*
**Process indicators, median (range)**			
AMS strategies	1 (1-2)	2 (0–2)	0.405
Monitoring of adherence to antimicrobial policy/treatment guidelines	1	1 (0.5-1)	**0.046**
Monitoring of antimicrobial usage	2 (0–2)	2 (0–2)	0.238
Surveillance of antimicrobial resistance (AMR)	2 (1-2)	2	0.317
Regular feedback to clinicians	2 (0–2)	2 (0–2)	0.157
Education on AMS	1	1 (0–1)	0.157
*Overall process indicator score*	*8* (6-10)	*9 (5-10)*	*0.246*
**Total score, median (interquartile range)**	*15 (10-19)*	*16 (7-19)*	*0.222*

^a^Differences investigated using Wilcoxon signed rank test

The following trust-level variables were also collected: ownership, highest level of care provided (secondary care, tertiary care, teaching, and specialized hospitals), number of beds, number of full time equivalent (FTE) dedicated infection control nurses per 100 beds.

Concerning outcome indicators, annual alcohol-based handrub usage and antimicrobial usage data, in terms of consumption of antibacterials for systemic use (ATC group J01), were collected for the years 2017–2021. Alcohol-based handrub usage was expressed as liters/1000 patient days (PDs), and systemic antibiotic consumption was expressed as defined daily doses (DDD)/1000 PDs. To reflect individual AMS strategies enacted in each trust, trusts could report antimicrobial usage data for a minimum of four antimicrobial classes, as long as the same classes were monitored each year. Annual antimicrobial resistance (AMR) rates for the years 2017–2021, in terms of methicillin-resistant *Staphylococcus aureus* (MRSA) and carbapenem-resistant enterobacteria (CRE, including carbapenem-resistant Acinetobacter spp., *Escherichia coli*, *Pseudomonas aeruginosa* and *Klebsiella pneumoniae* isolates) over all respective invasive isolates, were obtained from the regional surveillance system. The system applies European center for disease prevention and control (ECDC) European Antimicrobial Resistance Surveillance Network [17] protocol and definitions, and collects data on isolates from bloodstream and cerebrospinal fluid infections from reference laboratories in the region [10, 17]. 

### Statistical analysis

Descriptive statistics were used to summarize trust characteristics, AMS program scores, and outcome measures. Quantitative variables were summarized using medians and interquartile ranges (IQRs), due to non-normal distribution (Shapiro-Wilk test). Trusts were categorized according to their 2021 structure and process scores into the following groups: <25th percentile (P, i.e. low outliers), 25th -75th P, and > 75th P (high outliers). Differences between single items and overall AMS program scores prior to (2019) and during the pandemic (2021) were assessed using Wilcoxon signed rank tests. A linear regression model was used to assess whether the 5-year trends (2017–2021) in outcome measures in relation to structure and process scores were statistically significant. Significant trends (*p*-value for the regression coefficient ≤ 0.05) were described as increasing or decreasing. Compound annual growth rates (CAGR) for each outcome were calculated to illustrate changes in outcome rates over time. The CAGR corresponds to the mean annual change as a proportion of the value in the first year (2017). All analyses were performed using SPSS v. 27.0 (SPSS Inc., Armonk, NY).

## Results

All public trusts [18] and a small number of private institutions [3] provided data for this study. Descriptive characteristics of included trusts are presented in Table 2. The majority of participating trusts were public, with an even distribution between trusts providing secondary and tertiary-level care. The median number of FTE dedicated infection control nurses was above the minimum threshold of 1 FTE nurses per 250 beds recommended by the World Health Organization (WHO) 2019 Minimum requirements for infection prevention and control programmes; however, the number of infection preventionists did not reach the desirable rate of one per 100 beds, which was strongly supported by WHO experts considering the growing patient acuity and complexity, together with the increase in roles and responsibilities of infection control nurses [19]. 

**Table 2:** Characteristics of trusts participating in the study (*N* = 23), Piedmont, Northern Italy, 2021

Characteristic	Value
Ownership, n (%)	
Public	20 (86.96)
Private	3 (13.04)
Level of care	
Secondary	9 (39.13)
Tertiary	9 (39.13)
Teaching	3 (13.04)
Specialized	2 (8.7)
Number of beds, median (interquartile range, IQR)	439 (292–603)
Number of full time equivalent infection control nurses per 250 beds, median (IQR)	1.33 (1.03–1.6)

**Fig. 1:**
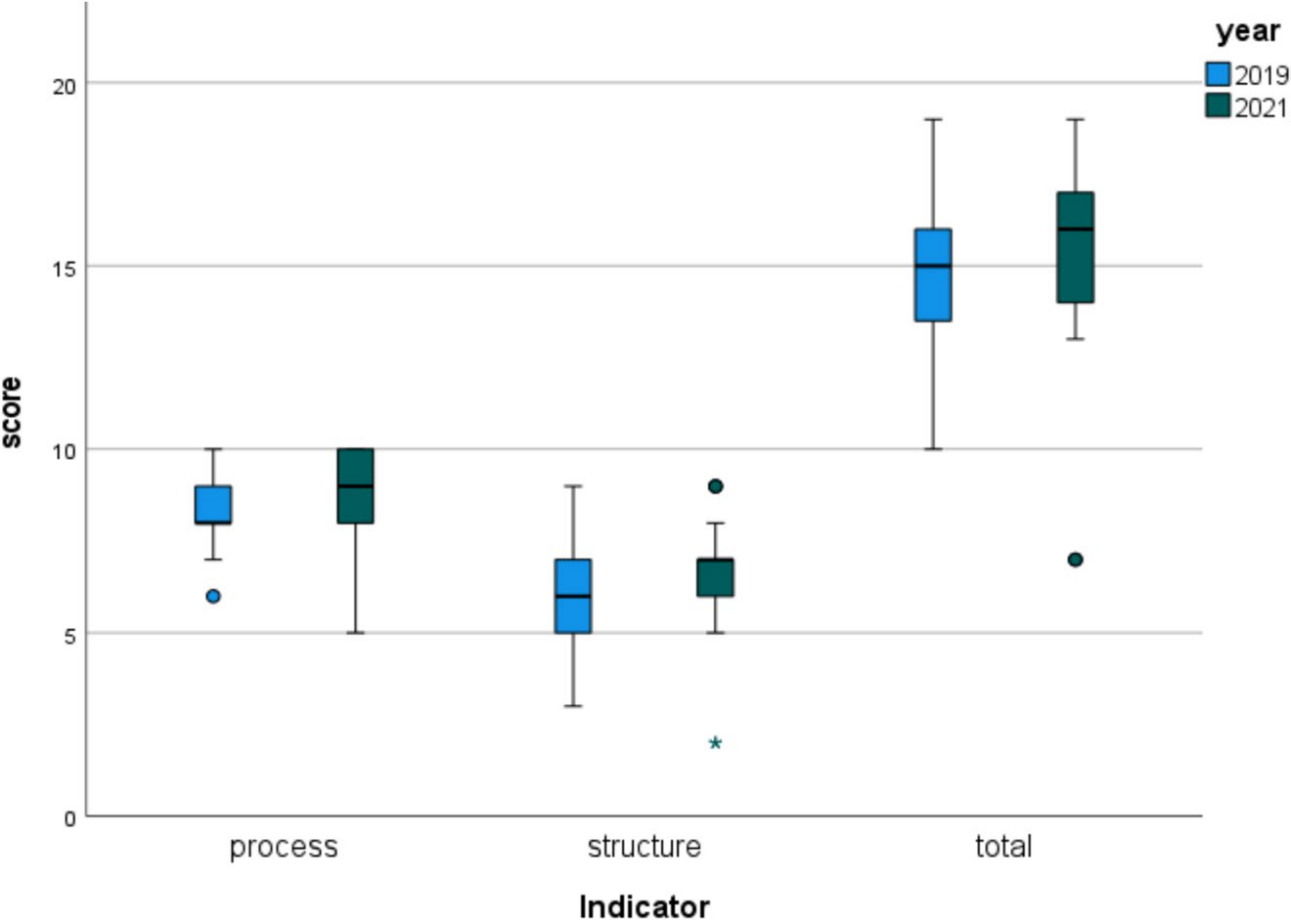
Box plots depicting structure, process and total scores assessed through quality indicators for the evaluation of antimicrobial stewardship (AMS) programs operating in the region of Piedmont, Northern Italy, 2019 vs. 2021 (*N* = 25 and *N* = 23 respectively)

Table 1 provides scores attributed to AMS programs in 2019 and 2021, according to the same quality indicator system. As shown in the table and in Fig. 1, a modest, non-significant improvement was found for 2021 structure, process, and total scores compared to respective 2019 scores. A significant improvement was found concerning the definition of a formal mission statement, whereas significantly less trusts included monitoring adherence to antimicrobial policy or treatment guidelines in their programs (22/23 in 2019 vs. 17/23 in 2021).

**Fig. 2:**
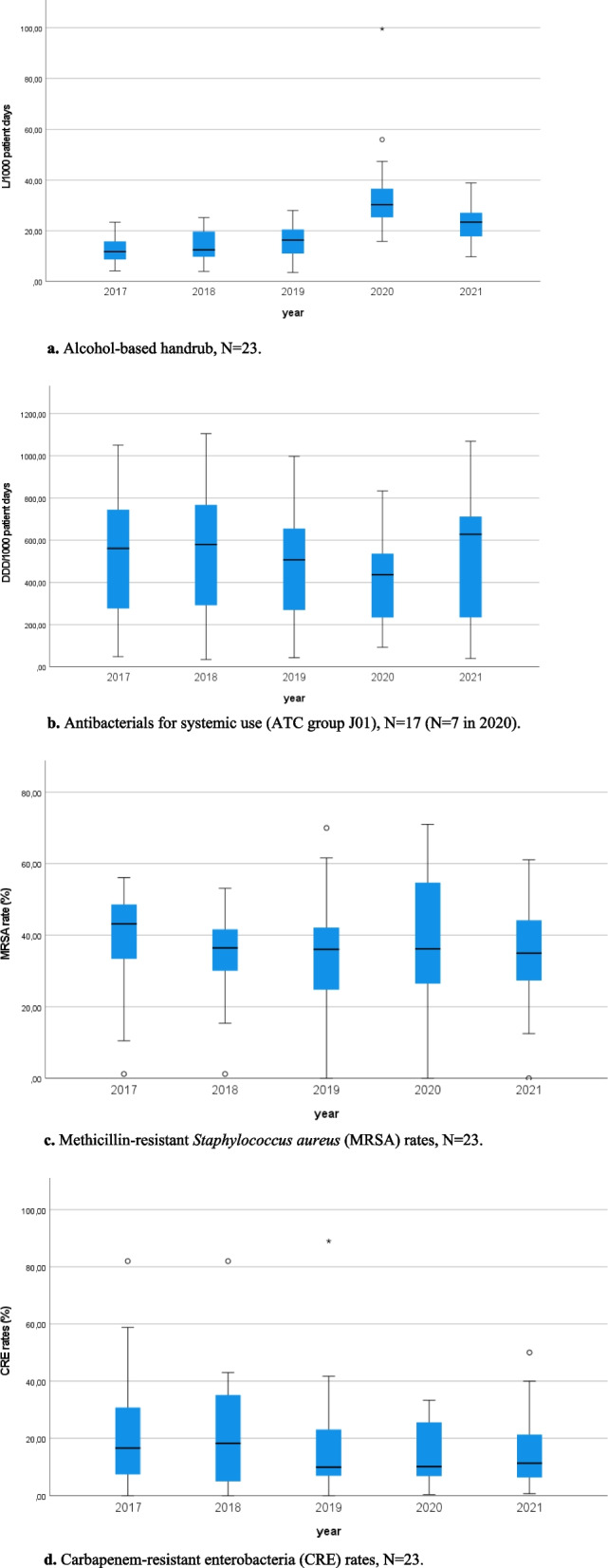
Outcome measures of trusts participating in the study stratified by year, Piedmont, Northern Italy, 2017-2021. **a.** Alcohol-based handrub, *N*=23. **b.** Antibacterials for systemic use (ATC group J01), *N*=17 (*N*=7 in 2020). **c.** Methicillin-resistant *Staphylococcus aureus *(MRSA) rates, *N*=23. **d.** Carbapenem-resistant enterobacteria (CRE) rates, *N*=23

Overall outcome measures per year are shown in Fig. 2; Table 3 reports trends in outcome measures stratified by quality indicator score groups. No significant trend was found for alcohol-based handrub usage, however, as shown in Fig. 2a, consumption increased over the five years with and overall median CAGR of 12.36% (IQR 9–19.95). Alcohol-based handrub consumption saw a peak in 2020, corresponding to the first pandemic year, and a slight decrease the following year. Concerning antibiotic usage, a significant downwards trend was found for trusts scoring between 25th – 75th P for structure indicators (Table 3); however, overall consumption saw an increase in 2021, with 2021 recording the highest median overall consumption compared to all previous years considered in this study (Fig. 2b). MRSA and CRE rates decreased over the 5-year period (Fig. 2c and d, median CAGR − 3.2%, IQR − 8.4 - -1.31, and median CAGR − 7.05, IQR − 11.75–2.87, respectively). Considering MRSA rates, significant downwards trends were identified for high-outlier structure and process groups. Stratifying trusts according to structure and total score groups, a pattern emerged for both MRSA and CRE rates, with greater CAGR reductions achieved over the considered period by groups with increasing scores (Table 3).

**Table 3:** Trends in outcome measures stratified according to antimicrobial stewardship (AMS) program quality indicators score percentile (P) groups of 23 trusts in Piedmont, Northern Italy, participating in the study (2017–2021)

Outcome measure	AMS structure score 2021			AMS process score 2021		Total AMS score 2021			All
	< 25th *P*	25th -75th *P*	> 75th *P*	< 25th *P*	≥ 25th *P*	< 25th *P*	25th -75th *P*	> 75th *P*	
**Alcohol-based handrub usage**									
5-year trend	NS	NS	NS	NS	NS	NS	NS	NS	NS
CAGR, median % (IQR)	10.27 (9–17.71)	12.93 (8.26–23.51)	10.69 (5.52–25.36)	11.32 (8.65–15.92)	12.36 (6.34–22.67)	9.98 (8.65–15.25)	12.93 (10.19–12.93)	8.17 (5.46–25.89)	12.36 (7.32–19.95)
**Antibacterials for systemic use (ATC group J01)** ^a^									
5-year trend	NS	**↓ (p 0.012)**	NS	NS	NS	NS	NS	NS	NS
CAGR, median % (IQR)	-2.99 (-6.07 - -2.99)	-0.46 (-3.76–3.97)	-2.07 (-5.86–7.01)	-0.46 (-6.07 - -0.46)	-1.88 (-3.57–1.03)	-0.65 (-6.07 - -0.65)	-2.25 (-3.57–0.21)	-0.42 (-7.2–9.89)	-1.17 (-3.18–1.92)
**Methicillin-resistant** ***Staphylococcus aureus*** **rates**									
5-year trend	NS	NS	**↓ (p 0.031)**	NS	**↓ (p 0.044)**	NS	NS	NS	NS
CAGR, median % (IQR)	-2.16 (-6.33–4.19)	-3.11 (-9.16–1.44)	-7.89 (-11.75–2.53)	8.53 (-2.16–8.53)	4.12 (-9.54 - -2.16)	-2.16 (-3.75 - -2.16)	-3.16 (-9.54 - -0.99)	-6.2 (-12.29–6.05)	-3.2 (-8.4 - -1.31)
**Carbapenem-resistant enterobacteria rates**									
5-year trend	NS	NS	NS	NS	NS	NS	NS	NS	NS
CAGR, median % (IQR)	0.26 (-7.17–7.47)	-6.47 (-18.79–16.5)	-10.77 (-27.59 - -3.4)	0.26 (-7.05–0.26)	-9.14 (-14.36–9.86)	2.87 (0.26–2.87)	-7.05 (-14.37–10.23)	-9.96 (-35.35 - -0.54)	-7.05 (-11.75–2.87)

^a^Antibiotic use data available from *N*=17 trusts (*N*=7 in 2020). *CAGR *Compound annual growth rate, *IQR *Interquartile range, *NS *Non-significant

## Discussion

Contrasting AMR is a globally recognized public health priority. One of the main drivers of AMR is inappropriate and excessive antibiotic use. Through public health initiatives and AMS activities, significantly decreasing trends in the consumption of antibiotics for systemic use (ATC group J01) have been registered across Europe during the past decade [20]. However, the disruption to healthcare activities caused by the recent COVID-19 pandemic led to important changes in prescribing patterns, patient case-mix, and hospital ecology. The reorganization of work activities, and the shift in priorities from contrasting AMR to preventing SARS-CoV-2 transmission caused difficulties in maintaining AMS, threatening the progress made [18, 21, 22].

Northern Italy was severely affected by the COVID-19 pandemic, and our region was part of the epicenter of the first pandemic wave [23, 24]. In this study, we aimed to investigate the impact of the pandemic on AMS programs and relevant outcomes from a healthcare system perspective, in a region highly endemic for AMR pathogens.

We found an improvement in the quality of AMS programs operating in our region between 2019 and 2021, measured through small increases in structure, process, and overall scores. These results were non-significant; however it is notable that AMS programs were not set back following the pandemic. In their survey of AMS programs implemented among a regional network of infectious disease units in Lombardy, Comelli et al. found 90% of included hospitals saw a reduction or temporarily suspension of activities during the first two pandemic waves, and less than 50% of units were able to restore pre-pandemic AMS programs immediately after [12]. 

Several characteristics of AMS programs in our region could have contributed to this result. First, the majority of AMS programs in Piedmont involve back-end or persuasive approaches, whereas restrictive strategies are applied rarely and only within combined strategies [13]. Even though back-end strategies have proven slower and less impactful in lowering antimicrobial consumption, several Authors have suggested they could be more acceptable by clinicians and more effective in improving prescribing appropriateness compared to front-end strategies, with longer-lasting and more sustainable effects over time [21, 25]. A previous study of an AMS program including prospective audits and feedback (PAF) at a single hospital of another northern region of Italy found resuming the program following the first pandemic wave could have contributed to the swift re-establishment of prescribing appropriateness [26]. In our region, significantly less trusts were able to maintain PAF during 2021 due to the amount of time and effort required for these activities, as found in other settings [22]. However, monitoring adherence was included in almost all evaluated AMS programs prior to the pandemic, which could have led to long-lasting results.

Second, our study found significant improvements in the number of trusts which had formally defined their mission statement, indicating enhanced commitment among all members of the AMS teams. In their survey, Comelli et al. found units of hospitals where management formally identified AMS as a priority objective were more frequently able to maintain their programs at or below pre-pandemic levels [12]. Finally, a strong quality infrastructure has been in place in Piedmont since 2008, and AMS programs have been promoted since 2012 within a regional framework. A qualitative study in UK hospitals found lack of proper guidance from public health authorities was an important challenge for AMS teams during the pandemic [22]. Results of another study set in a single Italian hospital suggest the long-term impact of AMS programs depends on its duration, as wards implementing AMS programs > 2 years did not register increased broad-spectrum antibiotic consumption during the considered pandemic period [21]. 

Concerning the outcome measures evaluated in this study, a general improvement was found in the direction of 2017–2021 trends: alcohol-based handrub consumption increased over the years, whereas antibiotic consumption and AMR rates generally decreased or remained unchanged. Significant downwards trends in MRSA rates in particular were found for high-outlier groups for both structure and process scores. For both MRSA and CRE rates, greater annual reductions were achieved by groups with increasing structure scores, in line with our previous findings of the importance of AMS program structure in improving relevant outcomes [13]. The COVID-19 pandemic affected outcome measures in different ways. Alcohol-based handrub consumption saw a peak in 2020, however this increase was not sustained through 2021. This finding is in line with the national trend and with previous reports [27, 28]. A global survey found many countries reported improvements in infection prevention and control activities resulting from the pandemic, including in terms of availability of alcohol-based handrub. However, inappropriate practices were also noted, due to healthcare workers focusing on self-protection over patient safety, namely double gloving, performing hand hygiene over gloved hands, and only performing hand hygiene during the final moments of patient care (after touching patients and their surroundings) [18, 29]. 

Following a progressive decrease through 2017–2020, the consumption of antibiotics for systemic use rebounded in 2021, rising to levels higher than all previous years considered in this study. The variations in antibiotic consumption rates seen during the pandemic years could be attributed to the important changes in healthcare delivery required for pandemic response. Due to the high toll of the first pandemic waves in Northern Italy, in particular in terms of patients requiring intensive care, most elective procedures were postponed, which significantly impacted patient case-mix [24]. The year 2021 saw a partial resumption of routine healthcare activities, however these were affected by the important back-log of procedures [18, 30, 31]. Further, high rates of inappropriate prescriptions, in particular of broad-spectrum agents, have been recorded among COVID-19 patients, such as for empirical coverage for possible respiratory tract co- or superinfections, and targeted treatment of hospital-acquired superinfections not limited to the respiratory tract [20, 32]. Both European and Italian national surveillance systems for antibiotic consumption highlighted an important increase in the proportion of broad-spectrum and last line antibiotic sub-groups over all antibiotics for systemic use, which reached 54.5% in Italy in 2021 and was generally much higher in Italy over the considered period compared to the European average [7, 20]. The high AMR rates in Italy could explain these differences in prescribing patterns, as seen in particular among healthcare-associated infections [6]. These results call for increased efforts to improve infection prevention and control and curb broad-spectrum antibiotic use. A multicentric cohort study in Michigan found some hospitals maintained low empirical antibiotic use among COVID-19 patients, suggesting well-structured and well-supported AMS programs can remain effective even in pandemic contexts [33]. 

In our study, the progressive decline in AMR rates did not appear affected by the pandemic. The European surveillance system for AMR recorded increasing CRE rates between 2017 and 2021. Conversely, European MRSA rates saw a progressive reduction, in line with our results [17]. According to national Italian data, MRSA and carbapenem-resistant *K. pneumonia* rates decreased in 2021 compared to 2019–2020, however increases were recorded for both carbapenem-resistant *P. aeruginosa* and Acinetobacter spp [10]. The aforementioned disruptions in healthcare delivery, as well as changes in behavior due to containment strategies, could have affected the risk of infection with AMR pathogens [17]. It must also be noted that decreases in requested cultures and in general in diagnostic capacity were noted globally, due to the diversion of staff, reagents and equipment to COVID-19 testing [18]. Further, it cannot be excluded that the impact on AMR rates will require more time to develop.

Several limitations of this study should be considered. First of all, AMS quality indicator scores are based on self-reported responses, which were not externally validated. Second, even though we received data from all public trusts in our region, only 7 trusts provided antibiotic consumption data for 2020 due to disruptions caused by the COVID-19 pandemic, limiting representativeness. Both antibiotic consumption and AMR data from 2020 should also be interpreted with caution due to changes in admitted patients and healthcare utilization, in particular reduced laboratory testing as previously mentioned. Outcome measures such as alcohol-based handrub use and antibiotic use, could also have been affected due to COVID-pandemic dynamics. Finally, several factors could have affected the impact of the pandemic on AMS programs and outcome indicators, therefore we make no claim of a causal relation.

## Conclusions

Despite these limitations, our study provided comprehensive data from a health system perspective, in a region highly endemic for AMR and severely affected by the pandemic. Results of this study suggest AMS programs in our region proved resilient to the challenges posed by the COVID-19 pandemic. This outcome was possible thanks to well-established programs, coordinated within a regional framework, the implementation of which requires considerable investment in time and resources. Characteristics linked to resiliency were suggested, however some areas for improvement were also highlighted. Continued efforts should be dedicated to supporting AMS programs and contrasting AMR, even when the focus is shifted towards other public health emergencies.

## Data Availability

The datasets used and/or analysed during the current study are available from the corresponding author on reasonable request.

## Abbreviations

AMR: Antimicrobial resistance
AMS: Antimicrobial stewardship
CAGR: Compound annual growth rate
COVID: 19–Coronavirus disease 2019
CRE: Carbapenem–resistant enterobacteria
DDD: Defined daily doses
ECDC: European center for disease prevention and control
FTE: Full time equivalent
HAI: Healthcare associated infection
IQR: Interquartile range
MRSA: Methicillin–resistant *Staphylococcus aureus*
NS: Non–significant
P: Percentile
PAF: Prospective audits and feedback
PDs: Patient days
PNCAR: Italian national action plan to contrast AMR
